# Identifying Key Target Audiences for Public Health Campaigns: Leveraging Machine Learning in the Case of Hookah Tobacco Smoking

**DOI:** 10.2196/12443

**Published:** 2019-07-08

**Authors:** Kar-Hai Chu, Jason Colditz, Momin Malik, Tabitha Yates, Brian Primack

**Affiliations:** 1 School of Medicine University of Pittsburgh Pittsburgh, PA United States; 2 Carnegie Mellon University Pittsburgh, PA United States

**Keywords:** smoking water pipes, waterpipe tobacco, tobacco, smoking, social media, public health, infodemiology, infoveillance, machine learning

## Abstract

**Background:**

Hookah tobacco smoking (HTS) is a particularly important issue for public health professionals to address owing to its prevalence and deleterious health effects. Social media sites can be a valuable tool for public health officials to conduct informational health campaigns. Current social media platforms provide researchers with opportunities to better identify and target specific audiences and even individuals. However, we are not aware of systematic research attempting to identify audiences with mixed or ambivalent views toward HTS.

**Objective:**

The objective of this study was to (1) confirm previous research showing positively skewed HTS sentiment on Twitter using a larger dataset by leveraging machine learning techniques and (2) systematically identify individuals who exhibit mixed opinions about HTS via the Twitter platform and therefore represent key audiences for intervention.

**Methods:**

We prospectively collected tweets related to HTS from January to June 2016. We double-coded sentiment for a subset of approximately 5000 randomly sampled tweets for sentiment toward HTS and used these data to train a machine learning classifier to assess the remaining approximately 556,000 HTS-related Twitter posts. Natural language processing software was used to extract linguistic *features* (ie, language-based covariates). The data were processed by machine learning tools and algorithms using R. Finally, we used the results to identify individuals who, because they had consistently posted both positive and negative content, might be ambivalent toward HTS and represent an ideal audience for intervention.

**Results:**

There were 561,960 HTS-related tweets: 373,911 were classified as positive and 183,139 were classified as negative. A set of 12,861 users met a priori criteria indicating that they posted both positive and negative tweets about HTS.

**Conclusions:**

Sentiment analysis can allow researchers to identify audience segments on social media that demonstrate ambiguity toward key public health issues, such as HTS, and therefore represent ideal populations for intervention. Using large social media datasets can help public health officials to preemptively identify specific audience segments that would be most receptive to targeted campaigns.

## Introduction

Hookah tobacco smoking (HTS)—also called waterpipe, shisha, or narghile—has increased substantially in popularity [1]. HTS is common among college students, with ever-use rates ranging from 30% to 46% [2,3]. It has been associated with multiple health conditions including cancer, cardiovascular disease, decreased pulmonary function, and nicotine dependence [4-6]. Owing to its high prevalence and deleterious health effects, HTS is a particularly important issue for public health professionals to address.

Social media sites can be a valuable tool for public health officials to conduct informational health campaigns. This process can be informed by machine learning approaches that are able to conduct topic classification [7] or sentiment analysis [8] in very large datasets. For example, researchers have also found a large amount of posted content on Twitter that describes HTS as positive [9] and normalizes the activity [10]. This is concerning because social media exposure to tobacco products is known to influence attitudes and future smoking behavior [11,12]. In response, public health departments have leveraged social media sites to conduct informational campaigns around HTS, including the Center for Disease Control and Prevention’s Tips from Former Smokers [13,14] and *ShishAware* [15].

Although programs such as these tend to use broad approaches, it may be more advantageous to tailor HTS-related educational messages to targeted groups or individuals [16]. Current social media platforms provide researchers with opportunities to better identify and target specific audiences and even individuals. However, we are not aware of systematic research attempting to identify audiences with mixed or ambivalent views toward HTS.

Therefore, this study is designed to accomplish 2 aims: (1) confirm previous research in HTS sentiment on Twitter [9,10] using a larger dataset by leveraging machine learning techniques and (2) systematically identify individuals who exhibit mixed opinions toward HTS via the Twitter platform. The latter procedure can provide actionable data for public health officials in developing educational campaigns for wide and efficient dissemination.

## Methods

### Data

Twitter is a microblog platform on which users post *tweets* that are shared either publicly or to their private network of followers. We collected 561,960 HTS-related tweets over 6 months, from January 1 to June 30, 2016. The search terms used were *hookah*, *hookahs*, *hooka*, *shisha*, *sheesha*, and *narghile*. We do not suggest that these 6 terms represent all possible HTS posted content; however, they follow previous research protocols [17] and allowed for collection of a large dataset of HTS tweets for our purpose. Data were collected by establishing connections to Twitter’s application programming interface, which permits external software to request data. All data collected were publicly available, that is, anyone with an internet connection was able to view the tweet at the time it was collected. Data included full text of the tweet content (maximum of 140 characters) as well as the identifier (ID) of the originating Twitter user to track individual users’ tweets over time. Tweet content was obtained in plain text format and reformatted by replacing *emoji* (images and symbols embedded within text) with human-readable counterparts (eg, a heart symbol becomes *[heart emoji]*) [17]. We also replaced specific hyperlinks and usernames with generic placeholders (eg, [URL]; @[USERNAME]). User IDs were then recoded to de-identified numeric IDs (eg, User 1). This study has been reviewed and approved by the Institutional Review Board at the authors’ university.

### Procedures

We applied machine learning algorithms to conduct sentiment analysis on HTS content posted to Twitter. Supervised machine learning allows for a relatively small amount of human-coded data to train computerized algorithms that can automatically categorize additional data on a scale that would not be feasible otherwise. We conducted the sentiment analysis in 2 phases. First, a set of approximately 5000 tweets were randomly sampled and categorized as being positive, negative, or both, and if it was commercial by 2 trained coders with experience in categorizing tobacco-related data on Twitter [17]. Inter-rater reliability measures for the human coders were all substantial or better [18], with Cohen kappa=.78 for positive/not positive and kappa=.75 for negative/not negative, and kappa=.82 for commercial. The coded tweets were subsequently used as training data for a classifier that automatically coded the sentiment in the remaining approximately 556,000 HTS-related Twitter posts. Natural language processing software RWeka and tm were used to extract linguistic *features* (ie, language-based covariates). The data were processed by machine learning tools in RTextTools.

In the second phase, human coders—informed by the previous classifications—identified Twitter accounts that had posted both positive and negative sentiment tweets about HTS, signaling a potential group of users with mixed or ambivalent views.

### Measures

Each tweet in the training dataset was given 3 categorical codes by 2 coders: (1) positive or not positive, (2) negative or not negative, and (3) commercial or not commercial. Commercial content was defined as anything promoting the sale of a particular hookah product, establishment, or related service (eg, a hookah bar promoting happy hour specials). These tweets were identified based on textual content rather than by the type of Twitter user posting the content (eg, a hookah bar could also post noncommercial content in other contexts; an individual user could promote a hookah bar). This allowed for us to maintain tweet content as the primary unit of sentiment analysis, rather than including user-level metadata (eg, establishment name or profile image) that our text-based machine learning classifier would be unable to judge. Sentiment (ie, positive and negative) was defined as *positive/negative toward HTS* rather than an overall positive or negative expression. This provided an advantage for supervised machine learning, as it allowed the classifier to be content specific, rather than depend on general sentiment terms. As previous research found that HTS-related content skewed positive [9,10], positive tweets were undersampled in the final training data to match the number of coded negative tweets.

### Analysis

To reduce the complexity of machine learning classifications, commercial tweets were included as positive or pro-HTS. After the machine learning completed classification of the approximately 556,000 tweets, 1000 tweets were randomly sampled to calculate 3 performance metrics: (1) precision, calculated as true positives divided by total instances labeled as positive; (2) recall, identical to sensitivity, calculated as true positives divided by total positive instances; and (3) F-score, a weighted average of precision and recall.

Finally, a qualitative analysis of Twitter users who had posted both positive and negative content was conducted. For this content search, tweets were grouped by user; based on an exploratory view by 2 authors of tweets in other topics (ie, beyond hookah-related posts), we decided that those with more than 5 times as many positive as negative posts, and vice versa, would not likely have truly mixed or ambivalent opinions and were removed from consideration. Coders were then tasked to identify potential users who had posted both positive and negative sentiment tweets.

## Results

**Figure 1:**
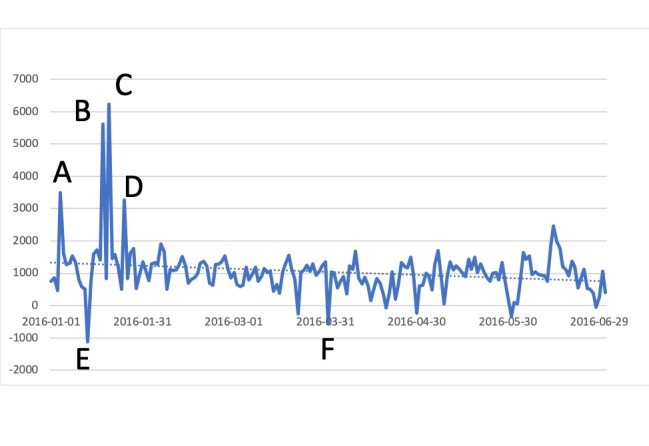
Sentiment of hookah tobacco smoking tweets over a 6-month period.

Table 1 provides the results of the full classifications, including summaries of precision and recall. Figure 1 shows the sentiment of HTS tweets over a 6-month period. Daily sentiment scores were calculated by subtracting the number of negative tweets from the number of positive tweets. These ranged from −1116 to 6239, with a mean of 1042 (SD 749).

There were 6 spikes during the 6-month period, defined as days where sentiment increased or decreased by more than 2 standard deviations. These are labeled A-F in Figure 1. The 4 positive spikes were as follows: (1) on January 4, a popular song about HTS (A); (2) on January 18, discussion of an HTS lounge (B); (3) on January 20, description of a concealable HTS device (C); and (4) on January 25, same as point 3 (D). The 2 negative instances were as follows: (1) on January 13, a report on the high levels of tar when using HTS (E) and (2) on April 1, an athlete was *caught* smoking from a hookah (F).

**Table 1:** Results of machine learning classifier with precision/recall metrics (January-June 2016).

Sentiment	Classified (N=561,960), n (%)	Precision	Recall	F-score
Positive	373,911 (66.53)	0.92	0.81	0.86
Negative	183,139 (32.59)	0.59	0.79	0.67

A total of 291,602 users posted HTS content over the period of the study, ranging from 1 to 6501 tweets each, with a median of 1. To identify users considered having ambivalent or mixed opinions about HTS, the criterion was defined as someone who had posted at least one positive and one negative tweet over the observed period. When we removed users that posted content with either positive or negative sentiment at a ratio greater than 5:1, 4.41% of users (12,861/291,602) remained. Of these users, we randomly sampled 1.00% of users (129/12,861) and selected all of their tweets to be qualitatively examined by 2 coders. There were 37 (29%) users classified as having clear ambivalent or mixed opinions about HTS. Examples of these users and their tweets are displayed in Table 2.

**Table 2:** A sample of 10 users (out of 37) who posted both positive and negative tweets about hookah tobacco smoking on Twitter between January and June 2016.

User	Tweet
1	Wednesday about to be lit lmao I need a hookah man^a^ I don't want hookah no more dawg lmao
2	Life feels so good when you are smoking hookah.. [blushing emoji]^a^ SO I TRIED VAPING TODAY, ON 108Hz.. HAHAHA fucking hard but such thick clouds, vaping is the best! gotta quit shisha and start vaping now!
3	@[USERNAME] stop smokin hookah then The hookah spot was rockin wit bitches feenin for cancer smh^a^
4	I'm smoking hookah in front of my building right now [URL]^a^ My goal is to not DJ any spots with Hookah this summer
5	Almost all my male friends love hookah smh^a^ Trying to put plans together for Chandra's birthday and I have to make sure hookah is involved [weary emoji]
6	Man y'all be paying 20 dollars at hookah spots to stare at each other [sobbing emoji]^a^ I only smoke shisha once in a while tbh lmao and wth we got jobs and school [URL]
7	She was sent from the heavens... She don't smoke hookah or know about lemonade. #Skinny I gotta find a way to make crab flavored hookah tobacco. #Skinny^a^
8	FAM be proud of me I havent smoked hookah ALL year -@[USERNAME] My ramadan nights bouta consist of me sitting on the porch till 5am skyping and smoking hookah.^a^
9	I wish hookah never existed [URL] There's no hookah so why go [URL]^a^
10	I've done hookah less than 5 times^a^ whenever I smoke hookah I wanna throw up

^a^Positive tweets.

## Discussion

### Principal Findings

This study combined several lines of research to integrate machine learning and online social media to inform public health research. We completed a 6-month sentiment analysis of HTS posts on Twitter and found that a majority (67%) of content posted about HTS were positive. This confirmed previous studies of HTS sentiment on Twitter with smaller datasets [9,10]. The 6 spikes in Figure 1 offered insight into how surveillance of HTS sentiment over time might be informative to public health departments. First, the identification of a positive spike following a newly released song referencing hookahs and the negative spike after a report of an athlete using a hookah demonstrated a capable process of having instantaneous access to public opinions of real-world events related to HTS. Immediate responses could be developed to address or leverage these situations, depending on the sentiment of the message. Second, reports of new HTS devices or technologies can also be quickly identified and allow for fast countermeasures to be taken as needed. Third, although the discussion of an HTS lounge also caused a positive spike, it was most likely to spread through use of automated bots or nonhuman accounts. Previous literature has found a significant number of Twitter accounts that discuss electronic cigarettes (e-cigarettes)—especially for advertisements—stemming from automated accounts [19]; it is possible a similarly high percentage are used for HTS as well. Additional research is needed to determine the prevalence of automated accounts that discuss HTS, as well as what effect this might have on the messages that are being spread.

Extending beyond a descriptive analysis of Twitter sentiment for surveillance, the second phase successfully identified users posting mixed or ambivalent sentiment tweets about HTS. The realization of our strategy was substantiated by 2 coders’ ability to detect several clear examples of posted tweets about HTS that differed in sentiment. We limited our discussion to 10 users for the sake of brevity, although more were discovered. These examples (Table 2) included people who had only tried HTS a limited number of times (users 6 and 10), people against HTS but who go to HTS lounges (user 3), people with lots of friends who use hookah (user 5), people trying to avoid or quit HTS (users 1, 2, 4, and 8), and people who recognize the potential negative side effects of HTS but still support or use it (users 7 and 9). These Twitter users could be an ideal audience for any public health campaign that is focused on HTS prevention or cessation. As the examples demonstrated, there was no clear pattern of vocabulary, topic, or other semantic features that were obvious in the data; instead, it was only by applying our method of aggregating tweets by users and then searching for mixed-sentiment content—from a dataset of approximately 561,000 posts—that we were able to identify these Twitter users. The ability to capture all of these users established the strength of the technique, as finding these users could not be easily accomplished manually.

Concerns exist when using machine learning to analyze topics with skewed data distributions; imbalanced data can reduce a machine learning algorithm’s capacity to properly classify data that are disproportionately small, also called a minority class. This is due to conventional algorithms being biased toward the majority class in an effort to optimize error rates [20]. For example, if a dataset of 100 tweets contained 90 positives and 10 negatives, a classifier that correctly labeled 83 positive tweets and 6 negative tweets would have an 89% accuracy; however, another classifier that labeled every tweet as positive would seem to score a *better* 90%, even though it was unable to detect any negative content. As previous research has shown that HTS-related content on social media tended to strongly be skewed positive, we chose a strategy to undersample positive tweets [21], trading decreased sensitivity for increased specificity with regard to the minority class. Similar results to previous studies combined with a reasonably high negative recall (Table 1) suggest that our approach was successful.

### Limitations

Limitations of our study include only using publicly available data from Twitter; inclusion of private Twitter content might lead to different results. Twitter user demographics can also limit the generalizability of these data. There is a possibility that a small percentage of HTS-related tweets are actually discussing using a hookah to smoke marijuana, although none were found in the sample that was human-coded. As we focused on linguistic features of the text, other media sources such as images or videos were not analyzed; expanding beyond linguistic features might also help improve the lower negative post precision. In addition, our strategy in choosing supervised machine learning restricts the results to HTS; a classifier would need to be retrained with content-specific data for other uses.

### Comparison With Previous Work

In recent years, public health officials have developed Twitter campaigns addressing tobacco products such as e-cigarettes. However, these campaigns can be hijacked by opposing organizations and result in countercampaigns. For example, the Chicago Department of Public Health released a series of messages about e-cigarettes a week before a scheduled vote on local regulation by the city council. Unfortunately, hundreds of tweets responded with opposing claims such as the health department was lying or disseminating propaganda [22]. In a similar fashion, the California Department of Public Health launched an anti–e-cigarette media campaign on Twitter called *Still Blowing Smoke*. As with the case in Chicago, a countercampaign quickly launched, called *Not Blowing Smoke*, that gained more attention [23]. In both cases, the original topics were meant to be general and far reaching; regrettably, those properties allowed for opposing organizations to have a single challenging message that could be disseminated quickly to the same population. Our study provides a unique method that generates data to identify precise subpopulations and specific topics that organically emerged from natural discourse. Having identified users with mixed opinions about HTS can be used as actionable data for a public health campaign. Rather than depend solely on expert knowledge to develop a single campaign that runs the risk of being misused by an opposing organization, this method provides the empirical evidence to build informational campaigns grounded on information that is actually needed by HTS users or groups.

### Conclusions

Tobacco control researchers focused on HTS should endeavor to develop campaigns that target this audience segment. Twitter has been proposed as a monitoring setting for public health [24,25], but the mixed success of other efforts to use mass user-generated data for large-scale public health detection [26] reveals the nonrepresentative nature of online and social media data in demographics and user patterns [27]. However, using social media as a setting to identify and reach potentially receptive audiences helps to avoid these threats to external validity. This mixed-sentiment strategy is not tobacco-specific and could be implemented in any public health setting. The approach could significantly improve the efficiency of any health campaign, allowing public health departments to be mindful with available resources and be confident of higher success rates.

Public health campaigns have frequently used mass media to disseminate educational or informational messages. State-level public health departments have also utilized Twitter to conduct informational tobacco campaigns with mixed results [22,23]. Although steps have been taken to leverage social media for these endeavors, officials have used strategies that do not always incorporate new types of information that technology can provide. We have demonstrated that user sentiment around HTS can and does change over time. Thus, it may be worthwhile to target public health interventions to individuals expressing positive or neutral sentiment toward HTS. Applying techniques in machine learning on large social media datasets can help public health officials to preemptively identify specific audience segments that would be most receptive to targeted campaigns. This allows a more purposeful and efficient method of producing changes in opinions, beliefs, or behaviors.

## Abbreviations

e-cigarette: electronic cigarette
HTS: hookah tobacco smoking
ID: identifier

## References

[1] (2016). Centers for Disease Control and Prevention.

[2] Barnett TE, Smith T, He Y, Soule EK, Curbow BA, Tomar SL, McCarty C (2013). Evidence of emerging hookah use among university students: a cross-sectional comparison between hookah and cigarette use. BMC Public Health.

[3] Primack BA, Shensa A, Kim KH, Carroll MV, Hoban MT, Leino EV, Eissenberg T, Dachille KH, Fine MJ (2013). Waterpipe smoking among US university students. Nicotine Tob Res.

[4] Haddad L, Kelly DL, Weglicki LS, Barnett TE, Ferrell AV, Ghadban R (2016). A systematic review of effects of waterpipe smoking on cardiovascular and respiratory health outcomes. Tob Use Insights.

[5] Kadhum M, Sweidan A, Jaffery AE, Al-Saadi A, Madden B (2015). A review of the health effects of smoking shisha. Clin Med (Lond).

[6] Sidani JE, Shensa A, Shiffman S, Switzer GE, Primack BA (2016). Behavioral associations with waterpipe tobacco smoking dependence among US young adults. Addiction.

[7] Cole-Lewis H, Varghese A, Sanders A, Schwarz M, Pugatch J, Augustson E (2015). Assessing electronic cigarette-related tweets for sentiment and content using supervised machine learning. J Med Internet Res.

[8] Myslín M, Zhu S, Chapman W, Conway M (2013). Using Twitter to examine smoking behavior and perceptions of emerging tobacco products. J Med Internet Res.

[9] Grant A, O'Mahoney H (2016). Portrayal of waterpipe (shisha, hookah, nargile) smoking on Twitter: a qualitative exploration. Public Health.

[10] Krauss MJ, Sowles SJ, Moreno MA, Zewdie K, Grucza RA, Bierut LJ, Cavazos-Rehg PA (2015). Hookah-related Twitter chatter: a content analysis. Prev Chronic Dis.

[11] Depue JB, Southwell BG, Betzner AE, Walsh BM (2015). Encoded exposure to tobacco use in social media predicts subsequent smoking behavior. Am J Health Promot.

[12] Yoo W, Yang J, Cho E (2016). How social media influence college students' smoking attitudes and intentions. Comput Human Behav.

[13] (2018). Centers for Disease Control and Prevention.

[14] Chung JE (2016). A smoking cessation campaign on Twitter: understanding the use of Twitter and identifying major players in a health campaign. J Health Commun.

[15] Jawad M, Abass J, Hariri A, Akl EA (2015). Social media use for public health campaigning in a low resource setting: the case of waterpipe tobacco smoking. Biomed Res Int.

[16] Thackeray R, Neiger BL, Keller H (2012). Integrating social media and social marketing: a four-step process. Health Promot Pract.

[17] Colditz JB, Chu K, Emery SL, Larkin CR, James AE, Welling J, Primack BA (2018). Toward real-time infoveillance of Twitter health messages. Am J Public Health.

[18] Cohen J (1960). A coefficient of agreement for nominal scales. Educ Psychol Meas.

[19] Allem JP, Ferrara E (2016). The importance of debiasing social media data to better understand e-cigarette-related attitudes and behaviors. J Med Internet Res.

[20] He H, Garcia EA (2009). Learning from imbalanced data. IEEE Trans Knowl Data Eng.

[21] Wallace BC, Small K, Brodley CE, Trikalinos TA (2011). Class Imbalance, Redux. Proceedings of the 2011 IEEE 11th International Conference on Data Mining.

[22] Harris JK, Moreland-Russell S, Choucair B, Mansour R, Staub M, Simmons K (2014). Tweeting for and against public health policy: response to the Chicago department of public health's electronic cigarette Twitter campaign. J Med Internet Res.

[23] Allem JP, Escobedo P, Chu KH, Soto DW, Cruz TB, Unger JB (2017). Campaigns and counter campaigns: reactions on Twitter to e-cigarette education. Tob Control.

[24] Lampos V, de Bie T, Cristianini N, Balcázar JL, Bonchi F, Gionis A, Sebag M (2010). Flu detector-tracking epidemics on Twitter. Machine Learning and Knowledge Discovery in Databases.

[25] Sadilek A, Kautz H, Silenzio V (2012). Predicting Disease Transmission From Geo-Tagged Micro-Blog Data. Proceedings of the Twenty-Sixth AAAI Conference on Artificial Intelligence.

[26] Lazer D, Kennedy R, King G, Vespignani A (2014). Big data. The parable of Google Flu: traps in big data analysis. Science.

[27] Blank G (2016). The digital divide among Twitter users and its implications for social research. Soc Sci Comput Rev.

